# The working experience of medical staff in the hospital‐wide bed‐sharing mode: A qualitative study

**DOI:** 10.1002/nop2.1940

**Published:** 2023-07-19

**Authors:** Ying Xiong, Juan Qin, Li‐li Zhou, Zhi‐feng Huang, Cai‐e Wu, Li‐ping Liu

**Affiliations:** ^1^ Department of Vascular Surgery The First Affiliated Hospital of Chongqing Medical University Chongqing China; ^2^ Department of Hematology Chongqing General Hospital Chongqing China; ^3^ Nursing Department Chongqing General Hospital Chongqing China

**Keywords:** bed management, hospital‐wide bed‐sharing, interdisciplinary admissions, medical safety

## Abstract

**Aim:**

The purpose of this study was to provide a comprehensive understanding of the attitudes and experiences of the medical staff regarding the hospital bed‐sharing model.

**Design:**

The present research was a qualitative study.

**Methods:**

This qualitative study used in‐depth individual interviews with 7 doctors, 10 clinical nurses and 3 head nurses, which were then transcribed and analysed thematically.

**Results:**

The study identified six overall themes. Issues were raised about the efficient utilization of hospital bed resources, greater challenges for nursing work, adjustment of doctors' work modes, barriers to communication between doctors, nurses, and patients, potential medical risks, and differentiation of patients' medical experience.

**Implications for Nursing Management:**

Hospital administrators and nurse managers should work together to solve the challenges that medical staff face, including strengthening nursing training, improving medical–nursing collaboration models, standardizing and effective communication strategies, and improving patient experiences.

## 1 INTRODUCTION

Medical services often limit resources (Ding et al., 2021; Li et al., 2021; Pu, 2021); thus, the rational allocation and efficient use of health resources have become an urgent problem in hospital management (Chan et al., 2015; Schmidt et al., 2013; Wu et al., 2020). Beds are one of the critical resources for in‐hospital emergency integration (Chartier et al., 2016; Cudney et al., 2019; Wong et al., 2010). However, both over‐utilization and under‐utilization of hospital bed resources occur worldwide (Li et al., 2021; Luo et al., 2019), resulting in a shortage of bed resources (Orsini et al., 2014; Zhou et al., 2020) in some departments, prolonged waiting times for hospitalization for patients (Bucci et al., 2016; McCarthy et al., 2009) and idle bed resources (Sheikhzadeh et al., 2012) in some departments.

As shown by several studies, bed‐sharing is an efficient way to utilize hospital bed resources (Franklin et al., 2022; Luo et al., 2019; Morris & Carter, 2015) and improve hospital inpatient flow (Claret et al., 2016; Jensen et al., 2012). The hospital bed‐sharing implies that the beds in each hospital ward are open to the entire hospital and that bed usage is managed centrally. The hospital conducts centralized management and unified deployment according to the admission needs. The number of beds is thus not restricted by department, and the doctor follows the patient. In addition to the clinical team provided by the original medical department, the hospital shares the nursing staff, beds, equipment, and other resources (Novati et al., 2017).

The value of hospital‐wide bed‐sharing has been demonstrated in research from other countries. American hospitals have established specialized Capacity Command Centers (CCCs) (Davenport et al., 2018; Franklin et al., 2022; Kane et al., 2019; Morris & Carter, 2015) to improve their overall perception and ability to coordinate actions and to carry out joint coordination among units in the hospital and even among hospitals. In German hospitals, patient admission planning and allocation are typically handled by the case management department (Case Management, CM) and the standard care department (Standard Care, SC) (Schmidt et al., 2013). The goals of the case management department are to improve all aspects of patient treatment, nursing and aftercare. Dutch hospitals have introduced bed classification based on length of stay, level of care and urgency (Bekker et al., 2017).

Although some research (Ajmi et al., 2022; Howell et al., 2008; Tortorella et al., 2013) has been conducted on this model, most studies have focused on benefit indicators such as the bed utilization rate and the average length of stay. There have been few studies on the cognitive attitudes of the medical staff, patients' experiences with medical treatment and unresolved issues in the implementation process.

The purpose of this study was to understand the cognitive attitudes and experiences of medical staff in a tertiary hospital in Southwest China towards the hospital‐wide bed‐sharing model, to explore further solutions to the issues in implementing this model, to promote the full utilization of medical resources and to address the ‘difficulty in seeing a doctor’ for patients. This question served as the starting point.

## 2 METHODS

### 2.1 Design

Since this study aimed to deeply understand the cognitive attitude and feelings of healthcare professionals participating in the hospital‐wide bed‐sharing mode, qualitative research using the phenomenological method was adopted.

### 2.2 Sample and setting

The front‐line clinical medical staff of a tertiary hospital in Chongqing was selected for interviews using the purposive sampling method. The study was conducted between September 2021 and March 2022 and included the analysis of various socio‐demographic factors such as departments, job divisions, working years, education and professional titles. Inclusion criteria: (1) the doctor/nurse had worked for more than 1 year; (2) the department/ward has participated in the hospital bed‐sharing model for over 1 year; (3) the study participant could express themselves well; (4) the study participant had submitted informed consent and participation was voluntary. Exclusion criteria: (1) participants on maternity leave, sick leave or going out for training; (2) if, over the previous 3 months, the total number of patients admitted across departments in the department/ward was less than 30 person‐times.

The interviewees provided informed consent to participate in the interview and agreed to have the entire process recorded. The sample size was determined by assuming that no new topics emerged during the interviews and that the data had reached saturation. Finally, 20 medical staff members were interviewed. Among them, there are seven doctors (D1–D7), two females and five males, aged 28–42 (35.71 ± 6.24) years, with working experience of 3–19 (11.71 ± 6.53) years, and 10 clinical nurses (N1–N10), all women, aged 26–40 (32.40 ± 4.43) years, with working experience of 3–20 (10.60 ± 5.21) years. There were three head nurses (HN1–HN3), all female, aged 26–40 (39.00 ± 2.65) years old, who had worked for 3–20 (18.67 ± 5.63) years. Table 1 depicts the characteristics of the interviewees.

**TABLE 1:** Characteristics of the interviewees (*n* = 20).

Serial number	Number	Gender	Age (year)	Department/ward	Education	Title	Position	Years of working (year)	Marital status
1	D1	Female	28	Oncology	Master's degree	Junior title	Doctor	3	Unmarri
2	D2	Male	28	General surgery	Master's degree	Junior title	Doctor	3	Married
3	D3	Male	33	Gastroenterology	Master's degree	Intermediate title	Doctor	16	Unmarri
4	D4	Male	42	Neurology	PhD	Deputy senior title	Doctor	16	Married
5	D5	Male	42	Department of Respiratory Medicine	Master's degree	Deputy senior title	Doctor	19	Unmarri
6	D6	Female	36	Oncology	Master's degree	Intermediate title	Doctor	10	Married
7	D7	Male	41	Otolaryngology	Master's degree	Deputy senior title	Doctor	15	Married
8	N1	Female	32	Joint spine ward	Undergraduate graduation	Intermediate title	Clinical nurse	10	Married
9	N2	Female	27	General/Oncology ward	Undergraduate graduation	Junior title	Clinical nurse	5	Married
10	N3	Female	31	Trauma ward	Undergraduate graduation	Junior title	Clinical nurse	8	Unmarri
11	N4	Female	32	Trauma ward	Undergraduate graduation	Intermediate title	Clinical nurse	12	Married
12	N5	Female	33	Joint spine ward	Undergraduate graduation	Junior title	Clinical nurse	11	Married
13	N6	Female	40	Urology ward	College graduation	Intermediate title	Clinical nurse	18	Married
14	N7	Female	33	Urology ward	Undergraduate graduation	Junior title	Clinical nurse	10	Married
15	N8	Female	26	Cardiac surgery ward	Undergraduate graduation	Junior title	Clinical nurse	3	Unmarri
16	N9	Female	39	Joint spine ward	Undergraduate graduation	Deputy senior title	Clinical nurse	20	Married
17	N10	Female	31	Cardiac surgery ward	Undergraduate graduation	Intermediate title	Clinical nurse	9	married
18	HN1	Female	40	General/Oncology ward	Undergraduate graduation	Intermediate title	Head nurse	18	Married
19	HN2	Female	41	Cardiac surgery ward	Undergraduate graduation	Deputy senior title	Head nurse	24	Married
20	HN3	Female	36	Gastroenterology	Undergraduate graduation	Intermediate title	Head nurse	14	Married

### 2.3 Data collection

The interview outline was developed through a literature review and group discussions. The interview outline mainly includes the following questions (Table 2).

**TABLE 2:** Interview schedule.

Interview schedule
1. Currently, the hospital implements a hospital‐wide bed‐sharing model, and our department participates in it. What do you think of this? 2. How do you feel about working in this mode? 3. You work in this mode. Have you encountered any problems related to this mode? What exactly is the problem? 4. What are the possible reasons for these problems? How did you solve it? 5. Have interdisciplinary patients expressed their feelings to you? How are they feeling?

Two researchers conducted semi‐structured face‐to‐face, in‐depth interviews with the respondents. The researcher made an appointment with the interviewee in advance. The interview time was arranged after the interviewee's daily work and was held in a quiet and comfortable study room of the department with recording equipment. The interview lasted approximately 30–45 min. Before beginning the formal interview, the researcher introduced himself, explained the purpose, main content, and significance of the interview, collected general information about the interviewee, promised to protect the interviewee's privacy, replaced the name with a serial number, explained the principle of recording, and obtained information about the interviewee. The visitor then agreed. One researcher conducted the interviews during the research, while the other recorded the interviews. During the interview, the interviewer listened carefully, maintained a neutral attitude and used appropriate interview techniques. The recorder noted the interviewee's tone, demeanour, mood, and movement changes and recorded them in the memo.

### 2.4 Ethical considerations

After selecting the participants at the beginning of the interview, the aims of the study were explained to them, and their verbal and written informed consent was obtained to participate in the study and for the audio recording. Participants were assured that the recordings would be used anonymously transcribed, their names would not be mentioned in any of the publications and the recorded audio would be deleted after the conversation resulted from the study. Participants were also given sufficient information regarding the review of data by other researchers to confirm the findings while maintaining confidentiality. Participants were free to refuse or to continue the interview, and there was no harm or loss to them. The project was approved by the Institutional Ethics Committee of Chongqing General Hospital (Project Identification Code: KY S2022‐083‐01).

### 2.5 Data analysis

Within 24 h after the interview, the recording was transcribed into text data, and the interviewee's tone, expression, demeanour, movement and emotional changes were marked in the corresponding position. Colaizzi's seven‐step analysis method (Lee, 2021) was used for data analysis using the following information: (1) detailed records and careful reading of all interview materials; (2) extraction of statements that were meaningful and consistent with the research; (3) summarizing and refining meanings from meaningful statements; (4) searching for common features or concepts of meaning to form themes and theme groups; (5) connecting the subject with the research and providing a complete description; (6) integration of the results by describing the research phenomenon in detail, and stating the essential structure of that research; and (7) feeding the results back to the research subject and asking them to confirm, thus improving the validity of the study. Two researchers independently analysed, coded and transferred the original data. If there was a disagreement, the research group discussed it and decided on a solution. We used consolidated criteria for reporting qualitative studies (COREQ) checklists for methodological quality assessment.

## 3 RESULTS

Through our analysis, we identified six overall themes, efficient utilization of hospital bed resources, greater challenges for nursing work, adjustment of doctors' work modes, barriers to communication between doctors, nurses, and patients, potential medical risks, and differentiation of patients' medical experience (Table 3).

**TABLE 3:** The working experience of medical staff in the hospital‐wide bed‐sharing mode.

Themes	Subthemes
Efficient utilization of hospital bed resources	The utilization rate of hospital beds was high
	Filling most beds
	The waiting times for patients to be admitted and for surgery have been reduced
Nursing faces greater challenges	
Nursing faces greater challenges	Higher expectations for our general nursing expertise
	Affected our communication and coordination skills
	Capability and resilience have also become more important
Increased demand for knowledge acquisition	Want to know more
	Requires a more comprehensive professional knowledge
	Learn and improve yourself
Increased workload of nurses	The frequency of patients being transferred has increased
	Frequently contact medical staff in multiple departments
	Spend time to learn will rise
The increased work pressure on nurses	Much to learn
	Lack self‐confidence
	A lot of pressure
The working mode of doctors has been adjusted	
Increased workload of doctors	Increases workload
	Need to run around
	Takes a lot of time
Higher requirements for doctors' sense of responsibility	Doctors need to proactively monitor the patient's condition
	Ensure medical safety and quality
	Take the initiative to provide some learning materials to the nurses for learning purposes
Barriers to doctor–patient communication	Could not solve problems promptly
	Making phone calls is the primary mode of communication
	Find the doctor takes a lot of time and energy
	Doctors and nurses are not as convenient and tacit
Potential medical risks	Unfamiliar with medications
	Difficult dealing with the doctor's orders
	Lack of relevant experience
	A lag in dealing with patients
Differentiation of patients' medical experience	Doctor is late coming to the ward
	Few opportunities to see doctor
	Inconvenient to find a doctor
	The ward environment is quiet and beautiful
	The nurses' skills are very good

### 3.1 Theme 1: Efficient utilization of hospital bed resources

The hospital‐wide bed‐sharing management model makes the hospital bed a shared resource for the whole hospital. The inpatients are arranged and allocated by the admission preparation centre to ensure the effective use of the beds in each ward and avoid the ‘imbalance of supply and demand, and different busy and busy schedules’ phenomenon, which improves the utilization rate of bed resources in each ward.

N4: ‘Before the implementation of hospital‐wide bed‐sharing, our department had very few patients, the utilization rate of hospital beds was not high, and beds remained vacant for a long time. After the implementation of this model, there are only a few vacant beds, and our income has also increased’.

HN3: ‘In the last 2 years, the number of patients in our department has increased, filling most beds. This model benefits us, particularly emergency patients who need surgery. For example, some patients with gastrointestinal bleeding may be transferred to another ward, and then the specialists of our gastroenterology department will monitor the patients so that they can receive timely treatment’.

D6: ‘Most of the time, our department has many patients. In the past, inpatient surgeries frequently required pre‐booking, especially during holidays when there were many students. After implementing bed‐sharing throughout the hospital, the waiting times for patients to be admitted and for surgery have been reduced’.

### 3.2 Theme 2: Nursing faces greater challenges

#### 3.2.1 Increased demands on nursing skills

The implementation of the hospital‐wide bed‐sharing management model was found to have increased the overall abilities of nurses and the levels of nursing professionals. Nurses require keen observational, communication, and coordination skills, and more comprehensive nursing skills, besides theoretical knowledge and business skills.

N9: ‘We had patients from most departments, simultaneously handling patients from seven different departments, leading to higher expectations for our general nursing expertise. At the same time, we had to communicate with doctors from other departments, which greatly affected our communication and coordination skills. Capability and resilience have also become more important’.

D4: ‘This paradigm has advantages, but it also places greater expectations on nurses, who must have a thorough awareness of diseases in many departments and master their care’.

#### 3.2.2 Increased demand for knowledge acquisition

Under the hospital‐wide bed‐sharing management model, nurses must master the knowledge and skills of their specialty while conversing with the knowledge and skills of other specialties resulting from interdisciplinary admissions. Thus, there is an increased demand for training.

N5: ‘I am a nurse who has worked in orthopedics for more than 10 years, but I am not experienced in observing and nursing patients who have undergone other types of surgery, such as endocrine surgery, breast surgery, and otolaryngology. I am not so confident. I want to know more about it. Training, for example: let us go to other departments to rotate learning’.

N8: ‘Working in this mode requires a more comprehensive professional knowledge, and you need to learn and improve yourself’.

HN1: ‘Our department admits many interdisciplinary patients, some of which we have never encountered before. We will consult relevant departments to learn their nursing routines or ask doctors for observations on key points’.

#### 3.2.3 Increased workload of nurses

The increased numbers of patients admitted and the diversity of disease specialties have led to an increase in the workloads of nurses, as evidenced by the need for communication with medical staff from multiple departments and the participation in training organized by hospitals and departments.

N1: ‘The frequency of patients being transferred has increased. Some patients need to be transferred from our ward to another after their condition worsens. Some patients are unwilling to be accommodated in other wards and must be transferred back. Some doctors want their patients to be accommodated in their own departments’.

N9: ‘When we had the most departments, we simultaneously admitted patients from seven departments. We must frequently contact medical staff in multiple departments if there is any doubt about the doctor's order or if the patient wants medical attention. For the doctors, collaboration in the department takes a long time’.

N8: ‘The content we will need to learn will rise as the scope of admission to the department broadens. The head nurse will use the morning meeting to learn collectively or publish content on the workgroup and the ‘317 Nursing’ APP to learn by themselves’.

#### 3.2.4 The increased work pressure on nurses

Due to their relatively weak knowledge of other specialties and lack of clinical experience in these specialties, the work pressure on nurses has increased. This has led to some interdisciplinary patients having poor experiences and experiencing negative emotions, resulting in further pressure on the nurses.

N1: ‘I feel my knowledge and skills are insufficient, and there is still much to learn’.

N2: ‘Compared with patients in the undergraduate department, I still lack self‐confidence in nursing when I face patients with complications that need specialized attention in other departments’.

N10: ‘Nurses are still under a lot of pressure. They sometimes cannot find a doctor quickly and cannot deal with the patient's affairs. The patient is emotional and complains to our nurses. We are also getting irritated’.

### 3.3 Theme 3: The working mode of doctors has been adjusted

#### 3.3.1 Increased workload of doctors

In the bed‐sharing mode, the patients under the charge of a doctor may be scattered over different wards. The doctor must follow the patient and attend different wards during rounds. This results in the need to travel physical distances between the patients with greater frequency and to take into account the time needed to move between patients in different wards.

D4: ‘Patients in our department are admitted to many wards, sometimes there are 5–6 wards, and we need to go to ward rounds one by one in these wards, which increases our workload’.

D7: ‘Patients in our department are spread across the hospital in different wards. Our doctors need to run around; some wards are far away, which takes a lot of time. Furthermore, the nurses in other wards have a lot of issues when they first start. They have to call and ask because they are not specialists, and some questions are unclear’.

#### 3.3.2 Higher requirements for doctors' sense of responsibility

In this mode, doctors decide whether patients can be admitted to other wards based on the patient's condition. This requires doctors to have systematic professional knowledge and varied clinical experience. Simultaneously, doctors need to be more proactive in communicating with nurses and inpatients in other wards to understand changes in the patient's condition, placing heavier demands on the doctor's sense of responsibility.

D1: ‘Currently, the morning shift handover charge is carried out collectively by specialized doctors and nurses in specialized wards. Thus, instead of participating in their specialized morning shift handover duties, responsible nurses for interdisciplinary hospitalized patients would proactively communicate unusual problems like low blood sugar or high body temperature to their specialized doctors, doctors need to proactively monitor the patient's condition’.

D7: ‘Our patients are located in other wards. In order to ensure medical safety and quality, our doctors will take the initiative to provide some learning materials to the nurses for learning purposes. We will take the initiative to inform the nurses if there are special observation points’.

### 3.4 Theme 4: Barriers to doctor–patient communication

Doctors only possess patients, not permanent beds, under the hospital‐wide bed‐sharing concept. Patients will follow doctors, and doctors will follow the patient to whichever nursing ward the patient is in. The patients will be moved from a more densely populated region to a more dispersed one. The interaction between doctors and nurses has also shifted from a one‐to‐one ‘linear’ relationship to a ‘network‐like’ relationship, which introduces new challenges to communication between doctors, nurses and patients.

N10: ‘Some interdisciplinary hospitalized patients complained that it was difficult to find a doctor and could not solve their problems promptly because their doctor in charge was not in the same nursing ward. Thus, the patients could only go to other wards to find a doctor or ask their ward to find a doctor. The nurse conveyed it to his doctor. When the doctor does rounds in the ward, he always checks on the patients in this ward before checking the patients in other wards. These interdisciplinary patients feel that the doctor is late coming to the ward’.

N1: ‘Our ward sometimes admits patients from 5 to 6 departments; thus, our nurses need to communicate with the doctors in these departments. Making phone calls is the primary mode of communication. No one answers the call; sometimes, the person who answers the phone is not the corresponding doctor in charge. Thus, it must be relayed, which is inconvenient, takes time and energy, and cannot fix the problem quickly’.

N2: ‘When we are on night shift, sometimes we cannot find the doctor in time. Although we have the phone number of their doctor's offices and nurse stations, the doctor must also care for patients in multiple wards. He also needs to go to the ward at night. Sometimes it is difficult to find a doctor using a public phone, and one needs to call the doctor's private number. But the doctors' duties are not fixed to a single ward; they roam, and finally, one needs to call the nurses in their departments, which is more troublesome’.

N3: ‘Communication between doctors and nurses across departments is not as convenient and tacit as communication between doctors and nurses in undergraduate departments. Therefore, we will establish a corresponding medical and nursing group for patients in which department we admit, including their specialist doctors and our nurses. Some of the issues are not urgent. The issue has been mentioned to the group and will be addressed when they have time’.

D7: ‘Our patients have been transferred to different wards. Our doctors are unable to interact with the nurses on certain units. We can only report to the shift in writing by phone or WeChat group if any matters need to be handed over’.

### 3.5 Theme 5: Potential medical risks

In the hospital‐wide bed‐sharing model, due to physical distance and the inexperience of nurses, there are certain risks in the processing of doctor's orders and drug use.

N2: ‘I am familiar with the usage of medications in my department, but I am unfamiliar with medications in other departments, and it is often difficult dealing with the doctor's orders. It is difficult to observe if the doctor has been careless and has prescribed the incorrect dosage or usage. Further, certain drugs pose a risk’.

N3: ‘Due to a lack of relevant experience, I do not know how to deal with unforeseen situations associated with multidisciplinary patients, such as unplanned extubation of some unusual pipelines. I do not know how to deal with them and pay attention to them, so I cannot be very good. I am dealing with it for the first time’.

N6: ‘There is a big difference in the use of drugs in each department. When encountering unfamiliar drugs, we have issues with their usage, precautions, and observation of adverse reactions’.

N7: ‘When we are looking for a doctor at night, and if the doctor does not leave his contact information and is not in the office, we need to call the nurse station to relay the information. It is quite troublesome, and there is a lag in dealing with patients’.

N9: ‘Sometimes there are special cases where the patient requires urgent attention. For example, a tumor patient has a burst of pain. The patient is in great pain and is anxious. He urges the nurse to give him painkillers, which are associated with some risk’.

### 3.6 Theme 6: Differentiation of patients' medical experience

Due to the different geographical locations of medical departments and nursing wards for interdisciplinary patients, some patients lack a sense of belonging and security while hospitalized. Simultaneously, some patients are more satisfied since the nurses are more skilled, the environment is better and the ward is affordable.

N1: ‘Some patients are disappointed since the doctor comes late for ward rounds, and there are few opportunities to see him’.

N7: ‘There are many interdisciplinary patients in our ward. Some patients complain that they cannot find a doctor or have trouble with a doctor, and their emotions escalate, affecting other patients, so they ask to be transferred back to his department. One patient felt that he had borrowed our hospital bed. He did not belong here and did not want to bother our nurses’.

D6: ‘There are patients in our department who wish to be accommodated in a VIP (very important person) single ward, but we do not have that. So, if her condition permits, she will reside in the VIP single room of the special needs ward, where the original doctor will diagnose and treat her, and she is quite pleased. There are also patients in their special‐needs wards who wish to be accommodated in a more economical ward and beg to be transferred to our ward’.

N2: ‘Our department has interdisciplinary patients who have long spent on our two‐person ward. One patient does not want to be transferred back to his ward. He thinks the ward environment is quiet and beautiful, the nurses' skills are very good. Some patients find it inconvenient to find a doctor, patients with good self‐care ability are better, and bedridden patients are more difficult’.

## 4 DISCUSSION

The primary reasons for the uneven use of bed resources include the division of departments and beds based on the type of disease (Boaden et al., 1999), relatively independent management of the beds in each department, opaque bed reservation information (Gopakumar et al., 2016) and limited energy of medical staff in bed management (Dreher, 1988; Virtanen et al., 2008). The unavailability of beds in departments with short supply leads to wait longer times (Ma et al., 2022), delayed antibiotic dosing, a higher likelihood of adverse events and a reduction of nurses' satisfaction (Blay et al., 2012; Hiragi et al., 2022), resulting in patients staying in emergency departments. In order to solve the shortage of hospital beds, some departments add beds at will, and the use and management of beds are not standardized, increasing medical risks (Maldonado et al., 2021).

In American hospitals, the bed‐sharing management approach is primarily concentrated in the emergency department (Davenport et al., 2018; Kane et al., 2019; Morris & Carter, 2015), where the scope of application is restricted. When assigning patients in German hospitals, implementing the bed‐sharing management approach cannot minimize the spatial distance from the treatment clinic. The development of management models may result in smaller hospital wards being exposed to the variability of the healthcare process.

The most prominent feature of bed‐sharing throughout the entire hospital is that specialized departments do not restrict the beds and are open to use by other departments within the hospital. This model breaks the traditional management model of one nursing team corresponding to one specialty and realizes that beds are no longer the resources of doctors. A centralized department allocates beds according to hospital beds and patients' conditions, and the nursing unit manages bed resources in a unified manner. The hospital‐wide bed‐sharing model avoids the blind expansion of the hospital scale. Under the premise of the existing moderate scale, limited medical resources can be allocated and used more reasonably, the management and work efficiency are improved, bed operation efficiency and space utilization are improved, and the service quality promotes the healthy development of the hospital (Abedian et al., 2014; Schmidt et al., 2013).

The present analysis showed the limitations associated with bed‐sharing throughout the hospital, and the following solutions are offered based on the interview results.

### 4.1 Enhance risk prevention awareness and ensure patient safety

The primary requirement is strengthening the training and analysis of the core system, supervising its implementation, and continuously improving the quality of medical care. This would enhance the system of reporting non‐punitive adverse events and encourage active reporting. The head nurse organizes and encourages all employees to participate in a discussion and analysis of the reasons for adverse events and the taking of corrective measures. The nursing department undertakes a retrospective examination of adverse events reported by the department regularly and suggests remedial solutions for existing problems and potential hidden dangers. A case database of typical adverse events should be established, together with conducting discussions and studies throughout the hospital, thus increasing awareness and predicting and preventing risks.

### 4.2 Strengthen nursing training and improve comprehensive ability

The ward training curriculum should be developed based on the categories of non‐specialized patients admitted to each nursing ward, and nurses should receive focused specialized knowledge and skills training. Simultaneously, critical thinking by nurses should be cultivated to address issues such as emergencies, communication, problem‐solving and professional confidence. Flexible and targeted training methods should be used in each ward based on the professional level and characteristics of the nurses, such as inviting nursing experts or doctors from relevant departments to give lectures, rotating training between nurses, nurses participating in doctors' rounds, and nurses participating in the teaching and training offered by related departmental organizations. New nurses have undergone two‐year standardized training, and the rotating departments include internal medicine, surgery, the emergency department and the operating room. Various types of training should be offered, such as online, offline and hybrid, so that nurses can flexibly arrange a time for learning. Each ward and nursing department should regularly organize relevant content assessments to inspect the training effects.

### 4.3 Establish an effective communication model and strengthen the communication between doctors, nurses and patients

When the admission preparation centre admits patients, arrangements are made based on the type of disease and proximity concept. Patients are told beforehand about the doctors' ward round arrangements and times. The doctor group conducts routine ward rounds twice daily in the morning and afternoon to promote communication between doctors and patients. This increases the doctors' sense of responsibility and encourages them to promptly interact with patients based on their condition and treatment plans. In order to facilitate rapid and effective communication, dedicated duty phones can be provided for duty doctors in various specialties, allowing duty nurses in each department to directly contact duty doctors. Communication training should be provided to nurses, particularly newcomers, to improve communication efficiency.

### 4.4 Improve the patient medical experience and improve patient satisfaction

When handling hospitalization, there should be full communication with patients and their families for those who can be admitted across departments. Doing so will help prevent patients from misunderstanding the hospital's procedures, leading to dissatisfaction and unnecessarily heated medical disputes. As the leaders of medical activities, doctors should take the initiative to strengthen communication with interdisciplinary hospitalized patients and solve patients' problems timeously. The professional and comprehensive training of nurses should be improved, allowing them to deliver more expert and better service to patients. The hospital should develop standardized health education manuals for each nursing unit to ensure that the nursing staff delivers health education effectively and to make it convenient for patients to learn about various diseases.

## 5 CONCLUSION

The hospital‐wide bed‐sharing model avoids the disadvantages of the coexistence of bed shortages and surpluses caused by the fixed bed management model, alleviates a series of problems such as difficulty in hospitalization, waste of medical resources, and potential safety hazards caused by extra beds, and avoids blind expansion of the hospital scale. The healthy development of the hospital is realized, and the independence of the nursing discipline is also reflected by raising the management level, increasing work efficiency, and making full use of the available bed resources. While this model allows efficient use of hospital bed resources, it also faces greater challenges in nursing work, adjustments to doctors' work patterns, communication barriers between doctors, nurses, and patients, potential medical risks, and differentiation of patients' medical experience.

### 5.1 Limitations of the study

The research subjects of this study were limited, as only medical professionals from a tertiary hospital in southwestern China were included, and studies with larger numbers of research subjects should be performed. Future research should also expand the survey parameters to allow in‐depth investigation of patients' and medical staff's satisfaction, further focus on potential medical risks, and conduct longitudinal research on patients' incidence rate and length of stay.

## AUTHOR CONTRIBUTIONS

The study design: LPL and YX; data collection: YX, CEW, LLZ and ZFH; data curation: YX; data analysis: YX and JQ; drafting of the manuscript: YX; supervision: LPL. All authors have read and agreed to the published version of the manuscript.

## FUNDING INFORMATION

No funding.

## CONFLICT OF INTEREST STATEMENT

The authors declare that they have no conflicts of interest.

## ETHICS STATEMENT

The study was approved by the Institutional Ethics Committee of Chongqing General Hospital (Project Identification Code: KY S2022‐083‐01).

## Data Availability

The data that support the findings of this study are available from the corresponding author upon reasonable request.
